# Endometrial cancer survival after breast cancer in relation to tamoxifen treatment: Pooled results from three countries

**DOI:** 10.1186/bcr3206

**Published:** 2012-06-12

**Authors:** Michael E Jones, Flora E van Leeuwen, Wilhelmina E Hoogendoorn, Marian JE Mourits, Harry Hollema, Hester van Boven, Michael F Press, Leslie Bernstein, Anthony J Swerdlow

**Affiliations:** 1 Section of Epidemiology, The Institute of Cancer Research, Sutton, Surrey, SM2 5NG, UK; 2 Department of Epidemiology, The Netherlands Cancer Institute, Plesmanlaan 121, 1066 CX Amsterdam, The Netherlands; 3 Department of Gynecology, University Medical Center Groningen, University of Groningen, Groningen, The Netherlands; 4 Department of Pathology, University Medical Center Groningen, University of Groningen, The Netherlands; 5 Department of Pathology, The Netherlands Cancer Institute, Amsterdam, The Netherlands; 6 Department of Pathology and Norris Comprehensive Cancer Center, University of Southern California Keck School of Medicine, 1441 Eastlake Ave, Ste 5409, Los Angeles, CA 90033, USA; 7 Division of Cancer Etiology, Department of Population Sciences, Beckman Research Institute, City of Hope, 1500 East Duarte Road, Duarte, CA 91010, USA

## Abstract

**Introduction:**

Tamoxifen is an effective treatment for breast cancer but an undesirable side-effect is an increased risk of endometrial cancer, particularly rare tumor types associated with poor prognosis. We investigated whether tamoxifen therapy increases mortality among breast cancer patients subsequently diagnosed with endometrial cancer.

**Methods:**

We pooled case-patient data from the three largest case-control studies of tamoxifen in relation to endometrial cancer after breast cancer (1,875 patients: Netherlands, 765; United Kingdom, 786; United States, 324) and collected follow-up information on vital status. Breast cancers were diagnosed in 1972 to 2005 with endometrial cancers diagnosed in 1978 to 2006. We used Cox proportional hazards survival analysis to estimate hazard ratios (HRs) and 95% confidence intervals (CI).

**Results:**

A total of 1,104 deaths occurred during, on average, 5.8 years following endometrial cancer (32% attributed to breast cancer, 25% to endometrial cancer). Mortality from endometrial cancer increased significantly with unfavorable non-endometrioid morphologies (*P *< 0.0001), International Federation of Gynaecology and Obstetrics staging system for gynecological malignancy (FIGO) stage (*P *< 0.0001) and age (*P *< 0.0001). No overall association was observed between tamoxifen treatment and endometrial cancer mortality (HR = 1.17 (95% CI: (0.89 to 1.55)). Tamoxifen use for at least five years was associated with increased endometrial cancer mortality (HR = 1.59 (1.13 to 2.25)). This association appeared to be due primarily to the excess of unfavorable histologies and advanced stage in women using tamoxifen for five or more years since the association with mortality was no longer significant after adjustment for morphological type and FIGO stage (HR = 1.37 (0.97 to 1.93)). Those patients with endometrioid tumors, who stopped tamoxifen use at least five years before their endometrial cancer diagnosis, had a greater mortality risk from endometrial cancer than endometrioid patients with no tamoxifen exposure (HR = 2.11 (1.13 to 3.94)). The explanation for this latter observation is not apparent.

**Conclusions:**

Patients with endometrial cancer after breast cancer who received tamoxifen treatment for five years for breast cancer have greater endometrial cancer mortality risk than those who did not receive tamoxifen. This can be attributed to non-endometrioid histological subtypes with poorer prognosis among long term tamoxifen users.

## Introduction

Tamoxifen is an effective treatment for breast cancer [1,2] but an undesirable side-effect is the increased risk of endometrial cancer in postmenopausal women [3-8], particularly rare tumor types [5,6,8,9] associated with poor prognosis [10]. Although the number of cases of endometrial cancer occurring after tamoxifen is modest (for example, 0.3% taking tamoxifen for approximately five years versus 0.1% not taking it [2]), there is concern that tamoxifen-induced endometrial cancers may have poorer survival [6,11], even after allowance for histopathologic characteristics [12]. The side-effects of tamoxifen are unlikely to outweigh the benefits in breast cancer patients [13], but any detrimental effects on survival would have implications for endometrial cancer surveillance following treatment [14], and would be important in decisions about the prophylactic use of tamoxifen by women without breast cancer [15]. To address these issues we have pooled patients from the three largest case-control studies of endometrial cancer after breast cancer [3-6] to examine mortality from endometrial cancer in relation to tamoxifen treatment.

## Materials and methods

The case series from three case-control studies of endometrial cancer after breast cancer were pooled. These studies from the Netherlands (NL) (nine regional cancer registries contributing to the Netherlands Cancer Registry), the United Kingdom (UK) (regional cancer registries in England, Scotland and Wales), and the United States (US) (Surveillance, Epidemiology and End Results (SEER) registries in four regions: Atlanta, Iowa, Los Angeles County, and Seattle-Puget Sound) have each been described previously [3-6]. Each study received appropriate ethical approval(s). The majority of data were abstracted from medical case-notes without patient contact; however, informed consent was obtained in the US where patients were interviewed. Briefly, each case-control study was population-based and included patients diagnosed with endometrial cancer after breast cancer during defined periods (NL (*n *= 765): 1978 to 1997; UK (*n *= 786): 1988 to 1996; US (*n *= 324): 1978 to 1993). The endometrial cancer diagnosis had to have occurred at least three months after the breast cancer diagnosis (six months for the US study). Patients were excluded if they had had a cancer (other than non-melanoma skin cancer or *in situ *cervical cancer) diagnosed before their breast cancer or between the diagnosis of the initial primary breast cancer and the subsequent endometrial cancer (except non-melanoma skin cancer, *in situ *cervical cancer or breast cancer). Information on tamoxifen treatment was abstracted from medical records and in Los Angeles, confirmed in interviews. At follow-up for survival, one patient from the original UK case-series was no longer eligible (because of erroneous cancer registry tumor record linkage) and was removed from this study.

The cases of endometrial cancer from the original Dutch study were supplemented with patients diagnosed from 1989 to 2003 (the TAMARISK (Tamoxifen Associated Malignancies: Aspects of Risk) retrospective cohort) [12] from the same nine regional cancer registries as in the original (ALERT (Assessment of Liver and Endometrial cancer Risk following Tamoxifen)) study [3,6], except diagnosis of endometrial cancer was at least 12 months after breast cancer (rather than three months). In addition, a further 179 Dutch patients diagnosed from 2003 to 2006 were included, with endometrial cancer at least three months after breast cancer, from the prospective component of the TAMARISK study [16].

### Follow-up

#### The Netherlands

Vital status, date of most recent follow-up, or date of death and cause, were obtained from medical records, general practitioners or clinicians, and municipal population registries. Follow-up for the ALERT patients was initially to 1997, with additional follow-up to 2004 for those patients who had less than four years initial follow-up. Follow-up was to 2003 to 2005 for the TAMARISK retrospective cohort and to 2004 to 2007 for the TAMARISK prospective cohort. All deaths were linked through 'Statistics Netherlands' [17] to obtain registered underlying cause of death (which was used in analyses when cause of death was unknown based on review of medical records [12]). Within the study period there were no known emigrations from the Netherlands in these cohorts.

#### UK

Vital status and cause of death were ascertained from hospital case-notes when the initial study data were collected (1996 to 1999). In 2005 further follow-up for vital status and causes of death was obtained from each of the regional cancer registries in Britain, and subsequently in 2008 further follow-up was obtained by linkage to the National Health Service Central Register (NHSCR -- a list of virtually every member of the population, which routinely receives notifications of events such as emigrations, cancers, and deaths) [18], and for those who had died copies of death certificates were obtained. Vital status could not be determined for eleven (1.4%) patients so for these follow-up was taken to the date of the last clinical contact as extracted from case-notes. Thirty-eight cases had deaths recorded as occurring at the date of diagnosis of endometrial cancer and were removed from the main analysis.

#### USA

Data were originally collected on vital status, date of most recent follow-up or date of death, and cause of death (based on information from death certificates) for all patients up to 2000. Additional follow-up was obtained to the end of 2006 for the Los Angeles County patients (*n *= 228), and those not known to be deceased were additionally checked against the Social Security Administration's Death Master File [19] to ascertain any deaths outside the state of California.

### Statistical Analysis

Descriptive analyses by morphological type of endometrial cancer were conducted using one way analysis of variance for continuous variables or Pearson chi-square for categorical variables [20]. When comparing individual differences between morphological groups, we adjusted for age at diagnosis of endometrial cancer and study, using linear regression in the case of continuous variables and a 'modified' Poisson approach with robust standard errors [21] for binary variables. To assess the association between tamoxifen treatment and the risk of death, we calculated hazard ratios using Cox proportional hazards regression [22] with time since diagnosis of endometrial cancer (follow-up time) as the implicit regression time scale and stratification by (adjustment for) attained age (which also is an adjustment for age at endometrial cancer diagnosis since: age at endometrial cancer diagnosis = attained age - survival time since diagnosis), calendar period and, as appropriate, morphology and FIGO stage. Tests for trend were calculated using continuous data. Women with deaths due to causes other than the cause under study in cause-specific analyses were treated as censored on their dates of death. Where it was not possible to distinguish between breast and endometrial cancer as cause of death the patients (*n *= 37) were not allocated to either cause of death in the main analyses, but were allocated to each cause in sensitivity analyses. Patients diagnosed with endometrial cancer at death (*n *= 38) were excluded from the main analysis and tables but were included, with a survival time of one day and one year, in sensitivity analyses. For breast cancer and all cause mortality, we additionally adjusted for age at diagnosis of breast cancer and extent of breast disease (instead of FIGO stage). All analyses were carried out using Stata/IC version 10.1 [23] and all statistical tests were two-sided.

## Results

### Descriptive characteristics of the three studies

There were 1,875 patients in the combined study, comprising 765 (41%) from the Netherlands, 786 (42%) from the UK, and 324 (17%) from the US (Table 1). The median age at diagnosis of breast cancer was 63 years in the Netherlands, 62 years in the UK study, and 65.5 years in the US study, and the median age at diagnosis of endometrial cancer was 69 years in each study. The calendar periods for diagnosis of breast cancer and endometrial cancer, and the intervals between the two cancers, reflect the original individual study designs (as described above). The median interval between cancers was 5.1 years in the Netherlands study, 6.0 years in the UK, and 3.0 years in the US. Tamoxifen use was more commonly recorded for patients in the UK (82%) than the Netherlands (46%) or US (45%).

**Table 1:** Characteristics of patients with endometrial cancer after breast cancer, by study

	Study						Total	
			
	NL		UK		US			
	
	N	%	N	%	n	%	N	%
Age at diagnosis of breast cancer (years)								
< 45	40	5.2	36	4.6	6	1.9	82	4.4
45 to 54	147	19.2	170	21.6	35	10.8	352	18.8
55 to 64	231	30.2	256	32.6	108	33.3	595	31.7
65 to 74	246	32.2	224	28.5	119	36.7	589	31.4
75 to 84	91	11.9	92	11.7	47	14.5	230	12.3
85 and over	10	1.3	8	1.0	9	2.8	27	1.4
Age at diagnosis of endometrial cancer (years)								
< 55	72	9.4	94	12.0	24	7.4	190	10.1
55 to 64	183	23.9	215	27.4	71	21.9	469	25.0
65 to 74	277	36.2	238	30.3	138	42.6	653	34.8
75 to 84	184	24.1	187	23.8	74	22.8	445	23.7
85 and over	49	6.4	52	6.6	17	5.2	118	6.3
Year of diagnosis of breast cancer								
1972 to 1979	37	4.8	60	7.6	33	10.2	130	6.9
1980 to 1984	98	12.8	196	24.9	122	37.7	416	22.2
1985 to 1989	163	21.3	376	47.8	147	45.4	686	36.6
*1990 to 1995*	238	31.1	150	19.1	22	6.8	410	21.9
*1995 to 1999*	152	19.9	4	0.5	0	0.0	156	8.3
2000 to 2005	77	10.1	0	0.0	0	0.0	77	4.1
Year of diagnosis of endometrial cancer								
1978 to 1984	16	2.1	0	0.0	38	11.7	54	2.9
1985 to 1989	63	8.2	112	14.3	143	44.1	318	17.0
1990 to 1994	141	18.4	501	63.7	143	44.1	785	41.9
1995 to 1999	257	33.6	173	22.0	0	0.0	430	22.9
2000 to 2006	288	37.6	0	0.0	0	0.0	288	15.4
Extent of disease (breast cancer)^a^								
Localized	362	47.3	317	40.3	198	61.1	877	46.8
Regional extension	295	38.6	150	19.1	121	37.4	566	30.2
Metastatic disease	15	2.0	4	0.5	5	1.5	24	1.3
Unknown	93	12.2	315	40.1	0	0.0	408	21.8
Interval between breast and endometrial cancers (years)								
< 1	35	4.6	47	6.0	34	10.5	116	6.2
1 to < 3	192	25.1	135	17.2	126	38.9	453	24.2
3 to < 5	150	19.6	134	17.1	73	22.5	357	19.0
5 to < 10	231	30.2	326	41.5	78	24.1	635	33.9
10 to 29	157	20.5	144	18.3	13	4.0	314	16.8
Morphological type of endometrial cancer								
Endometrioid adenocarcinoma^b^	636	83.1	666	84.7	278	85.8	1580	84.3
Serous or clear cell^c^	64	8.4	24	3.1	20	6.2	108	5.8
Carcinosarcoma^d^	37	4.8	56	7.1	15	4.6	108	5.8
Sarcoma^e^	26	3.4	19	2.4	9	2.8	54	2.9
Not known	2	0.3	21	2.7	2	0.6	25	1.3

All patients	765	100.0	786	100.0	324	100.0	1875	100.0

^a^Localized: no lymph node involvement; Regional extension: spread to lymph nodes; ^b^Endometrial adenocarcinoma, mixed cell adenocarcinoma, papillary endometrial adenocarcinoma; ^c^Serous adenocarcinoma, clear cell adenocarcinoma; ^d^Carcinosarcoma, Mullerian mesodermal mixed tumors; ^e^Sarcoma, endometrial stromal adenocarcinoma, leiomyosarcoma.

### Endometrial cancer morphology

In the combined series 60.7% of the endometrial cancers developed among tamoxifen users. The majority (84%) of the endometrial cancers were endometrioid adenocarcinomas (Table 2), and (after adjustment for study) these were diagnosed at significantly younger ages than were serous or clear cell endometrial cancer (*P *< 0.0001), and carcinosarcomas (*P *= 0.002). FIGO stage was available for 97% of the cases in the Netherlands, 78% in the US, but only 37% in the UK study. Where FIGO stage was known, 79% of tumors were stage I, 10% were stage II and 11% were stage III or higher, with no significant difference in this distribution between studies (*P *= 0.46). Endometrioid tumors were more likely to be diagnosed at FIGO stage I than were non-endometrioid tumors (*P *< 0.001). A significantly higher proportion of patients with carcinosarcoma had a history of tamoxifen use than did patients with endometrioid carcinoma (*P *< 0.001). Among tamoxifen users the patients who developed carcinosarcoma had been treated with tamoxifen on average 0.9 years longer than the patients with endometrioid type tumors (*P *= 0.012). Patients with carcinosarcomas, or serous or clear cell endometrial cancers, were more likely to have ceased tamoxifen use one or more years before diagnosis of endometrial cancer than patients with endometrioid tumors. (*P *= 0.010 and *P *= 0.020 respectively). The average interval between breast and endometrial tumors was longer for the unfavorable cancers, such as carcinosarcomas, serous and clear cell endometrial cancers but the differences were not statistically significant (*P *= 0.25).

**Table 2:** Age at diagnosis, tamoxifen use, FIGO stage, and interval between tumors, by morphology of endometrial cancer after breast cancer

	Endometrial cancer morphology^a^							
	
	Endometrioid carcinoma^b^		Serous or clear cell^c^		Carcinosarcoma^d^		Sarcoma^e^	
	
	*N *= 1,580		*N *= 108		*N *= 108		*N *= 54	
Age at diagnosis of endometrial cancer (years)								
< 55	165	10.4	4	3.7	8	7.4	12	22.2
55 to 64	413	26.1	19	17.6	17	15.7	15	27.8
65 to 74	553	35.0	39	36.1	39	36.1	17	31.5
75 to 84	363	23.0	34	31.5	33	30.6	9	16.7
85 and over	86	5.4	12	11.1	11	10.2	1	1.9
Mean (SD), years	68.9 (10.3)		73.4 (9.4)		71.2 (10.4)		67.6 (9.5)	
	analysis of variance (3 d.f.): *P *< 0.001^f^							
Tamoxifen use								
Not used	651	41.2	44	40.7	19	17.6	17	31.5
Used	929	58.8	64	59.3	89	82.4	37	68.5
	Pearson chi-sq (3 d.f.): *P *< 0.0001^g^							
Duration of tamoxifen use among users								
Used, < 2 years	272	29.3	19	29.7	11	12.4	10	27.0
2 to < 5 years	326	35.1	19	29.7	31	34.8	12	32.4
5 or more years	285	30.7	23	35.9	37	41.6	13	35.1
Used, duration unknown	46	5.0	3	4.7	10	11.2	2	5.4
Mean (SD), years	4.1 (3.2)		4.1 (2.8)		5.2 (3.3)		4.0 (2.8)	
	analysis of variance (3 d.f.): *P *= 0.025^g^							
Tamoxifen, time since last use among users								
Still on/≤ 3 months	649	69.9	34	53.1	50	56.2	24	64.9
3 months to < 1 year	70	7.5	5	7.8	5	5.6	3	8.1
1 year to < 3 years	73	7.9	8	12.5	10	11.2	6	16.2
3 years to < 5 years	35	3.8	6	9.4	7	7.9	2	5.4
5 or more years	56	6.0	8	12.5	8	9.0	0	0.0
Used, time unknown	46	5.0	3	4.7	9	10.1	2	5.1
Mean (SD), years	0.8 (2.0)		1.7 (2.8)		1.3 (2.2)		0.7 (1.3)	
	analysis of variance (3 d.f.): *P *= 0.002^g^							
FIGO stage								
I	914	57.9	46	42.6	35	32.4	21	38.9
II	111	7.0	14	13.0	3	2.8	4	7.4
III/IV	82	5.2	27	25.0	20	18.5	11	20.4
Unknown	473	29.9	21	19.4	50	46.3	18	33.3
	Peasron chi-sq (9 d.f.): *P *< 0.001^g^							
Interval between breast and endometrial cancer (years)								
3 to 12 months	102	6.5	8	7.4	3	2.8	2	3.7
1 to < 3 years	400	25.3	17	15.7	16	14.8	15	27.8
3 to < 5 years	305	19.3	18	16.7	18	16.7	12	22.2
5 to < 10 years	508	32.2	47	43.5	46	45.4	21	38.9
10 to 29 years	265	16.8	18	16.7	22	20.4	4	7.4
Mean (SD), years	5.9 (4.3)		6.5 (4.1)		6.9 (3.9)		5.2 (3.2)	
	analysis of variance (3 d.f.): (*P *= 0.039)^g^							

Total	1,580	100.0	108	100.0	108	100.0	54	100.0

^a^Excludes 25 patients where morphology was unknown; ^b^Endometrial adenocarcinoma, mixed cell adenocarcinoma, papillary endometrial adenocarcinoma; ^c^Serous adenocarcinoma, clear cell adenocarcinoma; ^d^Carcinosarcoma, Mullerian mesodermal mixed tumors; ^e^Sarcoma, endometrial stromal adenocarcinoma, leiomyosarcoma; ^f^adjusted for study; ^g^adjusted for age at diagnosis and study. N, number; SD, standard deviation.

### Follow-up

The 1,875 patients who had both breast and endometrial cancer were followed on average for 5.8 years (median 4.0 years) with 1,104 deaths (Table 3). For these patients with breast cancer who had also developed endometrial cancer 25% to 28% of the deaths were due to endometrial cancer, 32% to 35% to breast cancer (type of cancer death could not be distinguished between the two causes in 3% of cases), and 40% to all other causes (including 1.7% to cancer of unknown primary site and 0.5% with cause of death unknown). The five-year survival was 55.5% but this varied from 73% for patients diagnosed with localized breast cancer and FIGO grade I endometrial cancer to 16% for patients diagnosed with metastatic breast cancer or FIGO grade III/IV endometrial cancer. For those patients diagnosed with endometrial cancer before age 65, five-year survival was 82% for patients diagnosed with localized breast cancer and FIGO grade I endometrial cancer and 32% for patients diagnosed with metastatic breast cancer or FIGO grade III/IV endometrial cancer.

**Table 3:** Follow-up, vital status and cause of death for patients with endometrial cancer after breast cancer, by study

	Study						Combined	
			
	NL		UK		US			
Number of subjects	765		786		324		1875	
Total follow-up time (person-years)	2,676.6		5,552.2		2,731.5		10,960.3	
Maximum follow-up (years)	16.6		20.3		25.5		25.5	
Median length of follow-up (years)	2.6		5.2		8.1		4.0	
Vital status at end of follow-up (n, %)								
Alive	470	61.4%	202	26%	99	31%	771	41.1%
Deceased	295	38.6%	584	74%	225	69%	1,104	58.9%
Cause of death (n, %)								
Breast cancer	92	31%	198	34%	63	28%	353	32.0%
Endometrial cancer	85	29%	151	26%	41	18%	277	25.1%
Breast or cancer^a^	28	9%	9	2%	0	0%	37	3.4%
All other causes^b, c^	90	31%	226	39%	121	54%	437	39.6%
All causes	295	100%	584	100%	225	100%	1,104	100.0%
Survival (95% CI): all cause mortality								
1-year survival	87% (84%, 89%)		81% (78%, 83%)		87% (83%, 90%)		84.4% (82.7%, 86.0%)	
5-year survival	57% (53%, 61%)		51% (48%, 55%)		61% (56%, 66%)		55.5% (53.1%, 57.9%)	
Survival (95% CI): endometrial cancer mortality^d^								
1-year survival	93% (91%, 95%)		89% (87%, 91%)		94% (91%, 96%)		91.8% (90.5%, 93.0%)	
5-year survival	86% (83%, 88%)		81% (78%, 84%)		87% (82%, 90%)		83.8% (81.9%, 85.6%)	

^a^Not possible to differentiate between breast and endometrial cancer as cause of death; ^b^15 (2.6%) patients in UK and 4 (1.8%) in the USA study had cancer as cause of death, but primary site unknown; ^c^4 (1.4%) patients in NL and 2 (0.3%) in UK study had an unknown cause of death; ^d^deaths due to causes other than endometrial cancer are censored on date of death. CI, confidence interval; n, number.

### Mortality

#### Age at diagnosis of endometrial cancer

Older age at endometrial cancer diagnosis was associated with greater risk of dying of endometrial cancer (trend *P *< 0.0001, with no evidence for heterogeneity between studies (*P *= 0.52)) (Table 4). Subsequent analyses adjust endometrial mortality for attained age and time since diagnosis of endometrial cancer, and thus implicitly also adjust for age at diagnosis.

**Table 4:** Endometrial cancer mortality in relation to age at diagnosis, FIGO stage and morphology of endometrial cancer

	Patients	Endometrial cancer mortality		
	
	N	Deaths	HR	95% CI
Age at diagnosis of endometrial cancer^a^				
< 55	190	14	1.00	baseline
55 to 64	469	51	1.57	0.87, 2.84
65 to 74	653	87	2.13	1.21, 3.75
75 to 84	445	92	3.65	2.08, 6.43
85 and over	118	33	5.69	3.03, 10.7
Heterogeneity (4 d.f.)			*P *< 0.0001	
Trend (1 d.f.)			*P *< 0.0001	
FIGO stage^b^				
I	1016	64	1.00	baseline
II	132	26	3.34	2.11, 5.28
III/IV	140	67	13.1	9.25, 18.6
Unknown	587	120	2.92	2.06, 4.14
Heterogeneity (3 d.f.)			*P *< 0.0001	
Morphology^c^				
Endometrioid^d^	1580	162	1.00	baseline
Serous or clear cell^e^	108	32	2.25	1.51, 3.37
Carcinosarcoma^f^	108	54	5.41	3.92, 7.45
Sarcoma^g^	54	20	3.93	2.42, 6.38
Unknown	25	9	4.11	2.06, 8.14
Heterogeneity (4 d.f.)			*P *< 0.0001	
All non-endometrioid^h^	270	106	3.75	2.88, 4.87
Morphology by tamoxifen use^c^				
Tamoxifen users:				
- non-endometrioid	190	79	3.32	2.06, 5.35
-endometrioid	929	105	1.00	baseline
Tamoxifen non-user				
- non-endometrioid	80	27	3.88	2.86, 5.28
-endometrioid	651	57	1.00	baseline
Heterogeneity interaction^h ^(1 d.f.)			*P *= 0.57	
Morphology unknown:	25	9		

^a^Adjusted for time since diagnosis of endometrial cancer, study, calendar period, FIGO stage;

^b^adjusted for time since diagnosis of endometrial cancer, study, calendar period, attained age;

^c^adjusted for time since diagnosis of endometrial cancer, study, calendar period, FIGO stage, attained age;

^d ^endometrial adenocarcinoma, mixed cell adenocarcinoma, papillary endometrial adenocarcinoma;

^e^serous adenocarcinoma, clear cell adenocarcinoma; ^f^carcinosarcoma, Mullerian mesodermal mixed tumors; ^g^sarcoma, endometrial stromal adenocarcinoma, leiomyosarcoma; ^h^Excludes those where morphology was unknown. CI, confidence interval; FIGO, International Federation of Gynaecology and Obstetrics; HR, hazard ratio; N, number.

#### FIGO stage

Higher FIGO stage was associated with greater endometrial cancer death rates (FIGO III/IV versus I: Hazard Ratio, HR = 13.1; 95% confidence Interval (9.25 to 18.6); trend *P *< 0.0001 with no strong evidence for interaction between studies *P *= 0.067).

#### Endometrial cancer morphology

Endometrial cancer mortality was greater for patients with non-endometrioid endometrial cancer than patients with endometrioid types across all three studies combined (HR = 5.09; (3.96 to 6.53), *P *< 0.0001) and within each study (data not shown), with no evidence for heterogeneity between the three studies (*P *= 0.33); the greatest increases were seen for carcinosarcomas (HR = 6.66 (4.87 to 9.12)) and sarcomas (HR = 5.65 (3.53 to 9.05)). The HRs were smaller but still significant after adjustment for FIGO stage (Table 4).

#### Validity of cause-specific mortality

Extent of disease of breast cancer was unrelated to endometrial cancer mortality (*P *= 0.14) but was strongly related to breast cancer mortality (*P *< 0.0001). Age at diagnosis of endometrial cancer (*P *= 0.23), FIGO stage (*P *= 0.34) and endometrial morphology (*P *= 0.16) were not related to breast cancer mortality. Conversely, age at diagnosis of breast cancer was unrelated to endometrial cancer mortality (*P *= 0.11). There was no significant heterogeneity between studies.

#### Endometrial cancer mortality: tamoxifen use and morphology

No overall association was observed between tamoxifen treatment and endometrial cancer mortality (HR = 1.17 (95% CI: (0.89 to 1.55)); however, tamoxifen use for at least five years was associated with increased endometrial cancer mortality (HR = 1.59 (1.13 to 2.25)). After adjustment for morphological type and FIGO stage, five years tamoxifen use was no longer significantly associated with endometrial cancer mortality (HR = 1.37 (0.97 to 1.93)) overall or when stratified by morphology (Table 5). When analyzed by cumulative dose of tamoxifen, patients with cumulative doses over 30,000 mg (for example, 20 mg per day for 4.1 years) had modestly elevated endometrial cancer mortality. There was no association with daily tamoxifen dose.

**Table 5:** Endometrial cancer mortality and tamoxifen use, by morphology

	All morphological types				Morphology^a^							
					
					Endometrioid				Non-endometrioid^b^			
	
	d	n	HR^c^	95% CI	d	n	HR^d^	95% CI	d	n	HR^d^	95% CI
Tamoxifen use												
Not used	86	737	1.00	baseline	57	651	1.00	baseline	27	80	1.00	baseline
Used	191	1138	1.17	0.89, 1.55	105	929	1.01	0.72, 1.43	79	190	1.19	0.74, 1.89
	*P*-het (1 d.f.) = 0.26				*P*-interaction (1 d.f.) = 0.57							
Duration of tamoxifen use												
Not used	86	737	1.00	baseline	57	651	1.00	baseline	27	80	1.00	baseline
Used, < 2 years	29	313	0.71	0.46, 1.09	20	272	0.77	0.46, 1.29	8	40	0.50	0.23, 1.12
2 - < 5 years	61	397	1.18	0.84, 1.67	27	326	0.82	0.51, 1.32	30	62	1.64	0.94, 2.86
5 or more years	86	365	1.59	1.13, 2.25	49	285	1.43	0.94, 2.19	35	73	1.56	0.91, 2.69
Used, duration unknown	15	63	2.45	1.38, 4.36	9	46	3.14	1.50, 6.57	6	15	1.29	0.51, 3.26
	*P*-het (4 d.f.) = 0.0002				*P*-trend interaction (1 d.f.) = 0.36							
	*P*-trend (1 d.f.) = 0.0055				*P*-trend (1 d.f.) = 0.0023				*P*-trend (1 d.f.) = 0.22			
Cumulative tamoxifen dose (mg)^e^												
Not used	86	737	1.00	baseline	57	651	1.00	baseline	27	80	1.00	baseline
Used, < 7500	16	132	0.90	0.52, 1.55	12	114	1.06	0.57, 2.00	4	18	0.57	0.20, 1.65
7500 to < 15,000	15	159	0.72	0.41, 1.26	9	138	0.63	0.31, 1.29	5	20	0.83	0.31, 2.18
15,000 to < 30,000	32	239	1.06	0.70, 1.61	15	199	0.84	0.47, 1.51	15	35	1.38	0.72, 2.64
30,000 to < 60,000	56	294	1.40	0.97, 2.02	23	230	0.91	0.55, 1.51	31	59	1.64	0.94, 2.87
≥ 60,000	52	230	1.40	0.95, 2.05	32	185	1.29	0.81, 2.07	18	41	1.26	0.68, 2.36
Used, amount unknown	20	84	2.13	1.26, 3.58	14	63	2.86	1.54, 5.33	6	17	1.11	0.44, 2.81
	*P*-het (6 d.f.) = 0.022				*P*-trend interaction (1 d.f.) = 0.89							
	*P*-trend (1 d.f.) = 0.10				*P*-trend (1 d.f.) = 0.38				*P*-trend (1 d.f.) = 0.36			
Daily tamoxifen dose (mg/day)												
Not used	86	737	1.00	baseline	57	651	1.00	baseline	27	80	1.00	baseline
< 25 mg/day^f^	115	738	1.10	0.81, 1.50	63	610	0.97	0.66, 1.43	49	119	1.32	0.79, 2.20
≥ 25 mg/day^g^	62	343	1.25	0.88, 1.76	31	277	0.94	0.59, 1.48	27	59	1.11	0.64, 1.93
Used, dose unknown	14	57	1.74	0.96, 3.15	11	42	2.65	1.35, 5.20	3	12	0.72	0.21, 2.50
	*P*-het (*3 d.f*.) = 0.28				*P*-trend interaction (*1 d.f*.) = 0.90							
	*P*-trend (*1 d.f*.) = 0.86				*P*-trend (*1 d.f*.) = 0.68				*P*-trend (*1 d.f*.) = 0.60			
Time since last use (based on date of diagnosis of endometrial cancer)												
Not used	86	737	1.00	baseline	57	651	1.00	baseline	27	80	1.00	baseline
Still on/≤ 3 months	107	764	0.92	0.68, 1.26	62	649	0.82	0.56, 1.21	42	108	1.11	0.66, 1.88
3 months to < 1 year	14	87	1.12	0.63, 1.99	9	70	1.27	0.62, 2.62	4	13	0.64	0.22, 1.85
1 year to < 3 years	20	100	1.62	0.99, 2.67	7	73	0.90	0.41, 1.98	12	24	1.64	0.82, 3.30
3 years to < 5 years	13	53	1.91	1.05, 3.46	5	35	1.39	0.55, 3.50	6	15	1.82	0.73, 4.55
5 or more years	22	72	2.22	1.36, 3.61	13	56	2.11	1.13, 3.94	9	16	1.52	0.68, 3.38
Used, time unknown	15	62	2.11	1.19, 3.72	9	46	3.08	1.49, 6.38	6	14	1.23	0.49, 3.11
	*P*-het (6 d.f.) = 0.0007				*P*-trend interaction (1 d.f.) = 0.52							
	*P*-trend (1 d.f.) = 0.012				*P*-trend (1 d.f.) = 0.0033				*P*-trend (1 d.f.) = 0.20			
Interval between tumors												
3 - 11 months	9	116	0.83	0.40, 1.69	8	102	1.08	0.49, 2.38	1	13	0.19	0.02, 1.43
1 - 2 years	44	453	1.00	baseline	29	400	1.00	baseline	14	48	1.00	baseline
3 - 4 years	47	357	1.33	0.88, 2.01	22	305	1.02	0.59, 1.79	22	48	1.16	0.58, 2.33
5 - 9 years	110	635	1.62	1.13, 2.32	56	508	1.41	0.89, 2.23	52	117	1.31	0.71, 2.42
10 - 29 years	67	314	2.06	1.38, 3.08	47	265	2.18	1.35, 3.53	17	44	1.01	0.47, 2.16
	*P*-het (4 d.f.) = 0.0017				*P*-interaction (1 d.f.) = 0.13							
	*P*-trend (1 d.f.) = 0.0048				*P*-trend (1 d.f.) = 0.0003				*P*-trend (1 d.f.) = 0.49			

^a^Excludes those (9 endometrial deaths among 25 patients) where morphology was missing; ^b^Serous and clear cell, carcinosarcoma, sarcoma, excludes those where morphology unknown; ^c^Adjusted for time since diagnosis of endometrial cancer, study, calendar period, and attained age; ^d^Adjusted for time since diagnosis of endometrial cancer, study, calendar period, attained age, and FIGO stage; ^e^Trend evaluated on log10 transformed cumulative dose;

^f^Mostly 20 mg/day; ^g^Mostly 40 mg/day. CI, confidence interval; d, number of deaths attributed to endometrial cancer; HR, hazard ratio; n, number of patients.

Endometrial cancer mortality risk among women who stopped tamoxifen use at least five years before their endometrial cancer diagnosis was twice that of women who had never used tamoxifen and the trend with cessation among users (HR = 1.11 per year since last use (1.05 to 1.18)) remained statistically significant after adjustment for morphological type, duration of tamoxifen use, FIGO stage and interval between breast and endometrial cancer.

There was a strong trend of increasing mortality with increasing interval between breast cancer and endometrial cancer diagnosis (*P *= 0.0001), which, after stratification by morphology, remained statistically significant among those with endometrioid tumors (*P *< 0.0003). The trend with interval was also stronger in tamoxifen users than non-users (*P *= 0.044) and among users remained statistically significant even after adjustment for duration of tamoxifen use (*P *= 0.032). (For breast cancer mortality, risk of dying decreased as the interval between tumors increased (*P *= 0.003), but all-cause mortality did not vary with interval between tumors (*P *= 0.085)).

Time since last tamoxifen use and interval between diagnoses of breast and endometrial cancer are related (that is, only those with an interval between tumors of five or more years could have ceased tamoxifen use five or more years ago) but even among those patients with an interval of five or more years (that is, those with the potential for five or more years cessation) mortality was still elevated among those with five or more years since cessation of tamoxifen (HR = 2.06 (1.18 to 3.60)). When considering calendar period of diagnosis, since the indications for treatment and cessation of tamoxifen may have changed over time, there was no significant difference in trend with cessation among tamoxifen users for those diagnosed with breast cancer before 1990 (compared with those diagnosed in 1990 or later, *P *= 0.84) or with endometrial cancer before 1995 (compared with those diagnosed in 1995 or later, *P *= 0.53).

## Discussion

We accrued 1,875 patients, with 1,104 deaths of which 227 were due to endometrial cancer, by pooling the three largest case-control studies of endometrial cancer after breast cancer [3-6]. The number of cases of endometrial cancer occurring in breast cancer trial settings at present is modest (for example, 182 cases of uterine cancer reported in the Early Breast Cancer Trialists' Collaborative Group (EBCTCG) meta-analysis of 20 trials [2], and 102 cases in the National Surgical Adjuvant Breast and Bowel Project **(**NSABP) trial comparing prophylactic tamoxifen with raloxifene [24]), so although our data are observational we have a large number of cases, some of rarer histologies, with which to examine endometrial cancer survival after tamoxifen use. A previous report using a subset of the pooled data [12] showed increased endometrial cancer mortality with tamoxifen use, but did not have as many patients or as much follow-up time as we have in the pooled data.

We found that women with five or more years of tamoxifen use had 59% greater risk of endometrial cancer death than non-users, which was mostly attributable to the occurrence of endometrial cancer morphologies among tamoxifen users with less favorable prognosis, for example, carcinosarcomas. Several earlier studies have shown that tamoxifen greatly increases the risk of developing non-endometrioid tumors [5,6,8,9] and in the data reported here endometrial cancer mortality attributed to these morphological types was 2.3 to 5.4 times that of endometrioid types. Beyond the consequence of tamoxifen increasing the incidence of these tumors with poor prognosis [10], we saw no further adverse effect of tamoxifen dose or cumulative dose on endometrial cancer survival. In line with our results, genomic analyses suggest there are no differences between tamoxifen-induced tumors, either endometrioid or non-endometrioid, and those tumors occurring in patients without tamoxifen use [16,25,26]. Our data show, however, that patients with endometrioid tumors who had stopped tamoxifen five or more years before diagnosis of endometrial cancer had greater endometrial cancer-specific mortality risk. (The statistical power to examine this among patients with non-endometrioid tumors was low because these tumor types were less common.)

Although some of the women in this study may have received tamoxifen when distant metastases arose, and their prognosis would have been poor in relation to breast cancer survival, this does not preclude them from contributing survival time for analyses of tamoxifen use and endometrial cancer mortality (just as women who had other serious diseases are able to contribute to the analyses). As a demonstration of the validity of the cause-specific survival analyses, we found that extent of the breast disease was strongly predictive of breast cancer mortality but it was not associated with endometrial cancer mortality. Conversely, age at diagnosis of endometrial cancer, FIGO stage and morphology were strongly predictive of endometrial cancer mortality but not breast cancer mortality. We, therefore, believe our analyses are valid, whether tamoxifen was used for metastatic disease or in an adjuvant setting.

Mortality risk was elevated in the patients for whom tamoxifen use was known but the details of the dose or duration was missing. However, it is probable that this is an artefact related to the greater chance of destruction or loss of some part of the medical case-note history among patients who had died by the time of data collection. Our conclusions were not materially affected by the missing data because few were missing, for example, < 3% were missing the duration of tamoxifen use.

The cases from the three study populations were all ascertained from regional population-based cancer registries [3-6,12], although some patients were not available for the analyses. For the UK, 208 provisionally eligible patients were identified but their case notes could not be located or had insufficient information for the original case-control study (and subsequent follow-up for mortality). In the US, five patients were excluded from the case-control study. None were excluded in the NL, yet there was no evidence of heterogeneity between the studies, suggesting there was little, if any, bias due to case under-ascertainment. Furthermore, the one- and five- year survival rates seen here for endometrial cancer within each study were similar to published NL, UK and US rates [27,28], which suggests under-ascertainment did not materially affect the results.

Follow-up for mortality was comprehensive because population-based cancer registries covered each region, and additional information was available from medical records, general practitioners, clinicians, and national death registers. It is therefore unlikely that any significant migration outside of the study regions occurred, or that any unascertained deaths occurred. Thirty-eight patients diagnosed with endometrial cancer at death were excluded from the analysis because they contributed no survival follow-up, and their inclusion in sensitivity analyses made no material difference to the results. It was not possible to distinguish between breast and endometrial cancer as cause of death in 37 cases and these patients were censored at date of death in the main analyses, and their inclusion as endometrial cancer deaths strengthened the association with cumulative dose but made little material change to the other results.

An issue in the interpretation of the results is the attribution of cause of death to a single underlying cause in the presence of co-morbidity. We saw opposing trends in breast and endometrial cancer mortality with interval between tumors, and although longer follow-up since breast cancer would be expected to be associated with lower breast cancer death rates it is possible that deaths occurring after a long interval between tumors may have been more likely to be assigned to the most recently diagnosed tumor (that is, endometrial cancer). To make some allowance for this we adjusted for interval between tumors in the analyses and the main results remained the same: among patients with endometrioid tumors there was no association between endometrial cancer mortality and tamoxifen use, but increased mortality if tamoxifen had stopped at least five years before diagnosis of endometrial cancer.

Endometrioid endometrial tumors may be more likely to present with vaginal bleeding and therefore be diagnosed earlier than non-endometerioid tumors, and indeed we saw that these tumor types were more likely to be of a lower FIGO grade at diagnosis. However, even among the patients with only endometerioid tumors we saw increased mortality in those who had stopped tamoxifen five or more years before endometrial cancer diagnosis compared with those who had not received tamoxifen.

One possibility to consider is that gynecologic surveillance could have been less comprehensive after patients had ceased tamoxifen, resulting in delayed diagnosis and poorer prognosis. We investigated this hypothesis by looking at the effect of cessation among patients diagnosed with endometrial cancer before and after 1995 (when the first major reports of increased risk of endometrial cancer with tamoxifen use appeared [3,7,13] and awareness of the issue presumably increased), but we found that this did not change our findings, nor if we split the data at 1990 (when the first randomized trial results appeared linking tamoxifen to second cancers [29]).

If there is a real effect of time since last use we are unable to suggest an explanation for the increased risk but speculate that endometrioid endometrial cancers developing after long induction times may have different characteristics from those occurring in closer proximity to tamoxifen exposure.

## Conclusions

Patients with endometrial cancer after five years use of tamoxifen for breast cancer have increased mortality from endometrial cancer, due to the occurrence of less favorable morphological subtypes of endometrial cancer in long term tamoxifen users. Patients who had stopped tamoxifen use five or more years before diagnosis of endometrioid endometrial cancer had increased endometrial cancer mortality, a finding that warrants further research.

## Abbreviations

ALERT: Assessment of Liver and Endometrial Cancer Risk following Tamoxifen (a cohort study cancer in the Netherlands); CI: confidence interval; FIGO: Fédération Internationale de Gynécologie et d'Obstétrique (International Federation of Gynaecology and Obstetrics); HR: hazard ratio; NHSCR: National Health Service Central Register; SEER: Surveillance, Epidemiology and End Results; TAMARISK: Tamoxifen Associated Malignancies: Aspects of Risk.

## Competing interests

AJS holds shares in GlaxoSmithKline (who do not manufacture tamoxifen, but do make other drugs). The authors declare that they have no other competing interests.

## Authors' contributions

MJ, FvL, WH, LB and AS made substantial contributions to the conception, design, analysis and interpretation of data. All authors have been involved in acquisition of data, drafting the manuscript, revising it critically for important intellectual content, and have given final approval of the version to be published.
